# Self-Managed Leisure, Satisfaction, and Benefits Perceived by Disabled Youth in Northern Spain

**DOI:** 10.3389/fpsyg.2020.00716

**Published:** 2020-04-28

**Authors:** Joseba Doistua, Idurre Lazcano, Aurora Madariaga

**Affiliations:** Leisure Studies Institute Faculty of Social and Human Sciences, University of Deusto, Bilbao, Spain

**Keywords:** leisure, youth, disability, benefits of leisure, self-managed

## Abstract

Positive leisure is widely accepted as contributing to the development of self-autonomy and well-being of young people during their transition to adulthood (Glendenning et al., 2003; Coleman, 2011). However, there has been little research on these issues among young people with disabilities. In this study, we analyzed the relationship between self-managed leisure, satisfaction with leisure, and emotional, cognitive, and behavioral benefits as perceived by young people with disabilities. The sample consisted of 400 young people (48.8% female) with disabilities (hearing, physical, intellectual, and visual), aged between 15 and 29 years, who live in the Basque region of northern Spain. Results revealed the following conclusions. First, gender and type of disability relate to the degree of self-management associated with leisure. Second, there was a significant association between the degree of satisfaction with leisure and level of self-management associated with leisure and this relationship varied by disability type but not gender. Third, leisure independently organized by young people (self-managed) was associated with higher perceived psychological benefits (emotional and cognitive) connected with their leisure engagement.

## Introduction

Adolescence and early adulthood crucial and sensitive stages in the progression toward maturity. These are not only periods of rapid physical change but also important stages in personal, cognitive, and social development (Kristis, 2018). During youth, people explore their potential, develop diverse social roles, construct their personal identity, accept and reject habits, values and beliefs, socialize and build a lifestyle that typically remains, to some degree, for their entire lives (Berg et al., 2014; Mary, 2014; Gradaílle et al., 2016).

In addition to the family, other agents have an increasing impact on socialization, such as peer groups, the educational system, mass media, and social networks (Elzo and Silvestre, 2010; Aristegui and Silvestre, 2012). For some authors, the main agent of socialization is the peer group (Elzo, 2004; Rabino and Serra, 2017), they affirm that among young people and adolescents of the so-called post-modernity, in the occidental context, socialization is more carried out from group experimentation (sharing and rehearsing conducts and values) with other adolescents and young people and not so much from the reproduction of what has been transmitted by other historical instances of socialization such as family, school, churches, political parties and even media.

Leisure is one of the most important areas in young people’s lives. Fine et al. (1990) defined leisure as the social institution most closely associated with the world of adolescence and young people. Leisure is increasingly recognized as having a critical role in personal development during childhood and youth. It is an aspect of human development that offers anyone, including adolescents and young people opportunities to express themselves, demonstrate skills, and connect with others (Caldwell and Baldwin, 2003).

Numerous studies highlight the value of leisure for young people (e.g., Caldwell and Witt, 2011; Roberts, 2014; Gomez-Granell and Juliá, 2015). These experiences are beneficial because of opportunities for socialization and peer interaction. Leisure offers opportunities to experiment with diverse social roles, identification of individual preferences, exchange of experiences, development of skills to respond collaboratively to diverse situations, and development of friendships (Byrne et al., 2006). This period of life is particularly appropriate for discovering new interests and affirming personal values and other social ideals (Roult et al., 2014).

Within the growing body of literature in positive psychology, participation in leisure is recognized as providing opportunities for young people to engage in positive relationships, which helps in regulating emotional development, empathy, and prosocial self-efficiency (Osgood et al., 1996; Larson, 2000; McDonough et al., 2013). Thus, the participation in positive leisure offers young people potential for the exercise of self-determination (Anderson, 2017).

Experiencing positive emotions facilitates the construction of lasting personal resources, ranging from physical and intellectual resources to social and psychological resources (Fredrickson, 2001). Results of investigations suggest that leisure is associated with positive social and emotional development, and is part of the maturity process and increasing autonomy (Leversen et al., 2012; King and Church, 2015). In this sense, Caldwell and Smith (2006) suggest that, through participation in leisure, positive social interactions give young people the potential to develop skills and autonomy.

However, the transition from childhood to adolescence and young adulthood does not guarantee the capacity for participation and autonomy. According to Larson (2000), adolescents and young adults have limited opportunities to develop their decision-making skills due to the demands of school work and structured leisure organized by others, and therefore lack sufficient opportunities to develop autonomy. The most appropriate context for development of autonomy seems to be voluntary activities, experienced as leisure, such as sport, the arts, and participation in non-government or non-profit organizations.

During adolescence and youth, individuals are required to complete increasingly complex tasks and be involved in higher levels of decision-making (Eccles and Barber, 1999). Various studies (e.g., Flanagan et al., 1998; Flanagan and Gill, 1999) show how participation in community groups and institutions (e.g., school or youth organizations, and cultural, environmental, political, and religious communities) promotes social integration. Today, there is a wide range of practices and spaces in which youth can invest their time. However, this participation varies depending on the degree of autonomy, development of activity, or use of times and spaces. Thus, there coexists a gradation of activities that move from monitoring (under the supervision of a responsible adult) to self-management (Berrio-Otxoa et al., 2002; CEIC/IKI, 2005; Comas, 2011; Tejerina et al., 2012). Specifically, development of the young person relates closely to what some authors call the autonomous capacity for action (Ryan, 1993; Deci, 1995; Brandtstadter, 1998).

When young people and adolescents are very interested in leisure, their personal satisfaction grows. That is, an adolescent’s level of intrinsic motivation has a positively influence on satisfaction with leisure (Hills et al., 2000). Along the same lines, some studies indicate that young people report greater satisfaction with leisure in which they acquire greater autonomy and opportunities for self-management (Ortega et al., 2015). Thus, young people participate more and with higher levels of satisfaction in leisure they share with their peers and in which they assume higher levels of self-management or participation.

Many researches have examined benefits of leisure. Driver et al. (1991) defined benefits as changes seen as advantageous for improving the condition, an increase, or a progress, a change that is seen as advantageous for an individual, a group, society, or another entity. Driver and Bruns (1999) describe benefits of leisure in three ways: as improvement of a condition or situation in the framework of generative or proactive leisure; as prevention of an unwanted condition and/or maintenance of a desired condition in the context of a preventive or sustained leisure; and as attainment of satisfactory psychological experiences in the framework of an adjusted or autotelic leisure.

Different authors have studied the benefits of leisure and have organized and categorized them differently. However, most of them mention indicators related to physical benefits: improvement of physical condition, physical effort, physical well-being, new experiences and challenges, physical potential or being an active person (Gorbeña, 2000; Tinsley, 2005).

In addition, several studies describe aspects related to social benefits: interpersonal relationships and social skills or aptitudes, socialization processes, friendship, affiliation, behaviors that promote family and group functioning, group work skills (O’Morrow and Reynolds, 1989; Gorbeña, 2000; Tinsley, 2005; Barnett, 2013; Sibthorp et al., 2013).

In addition, there is research focusing on psychological benefits (O’Morrow and Reynolds, 1989; Tinsley, 2005; Sibthorp et al., 2013), whether they are behavioral (adjusted behavior, problem solving, perceived competence, desire to explore), cognitive (learning acquisition, challenges, new skills) and emotional, especially psychological well-being (Iso-Ahola, 2006; Trainor et al., 2010; Sonnentag, 2012; Sibthorp et al., 2013; Kim et al., 2016), as well as self-esteem, sensitivity, hedonism, and intrinsic reinforcement.

Research on perceived benefits or perception of benefits in the field of leisure focuses on a specific activity such as golf (Han et al., 2014) or those studies that have focused on specific population groups: young people (Oh et al., 2002); pregnant women (Da Costa and Ireland, 2013); adults (Stutts, 2002); older people (Dergance et al., 2003); or people with a disability such as spina bifida (Williams et al., 2014).

Positive effects of leisure on physical and mental health have been a consistent finding in different investigations. For example, Tinsley (2005) investigated how satisfaction of personal needs through participation in leisure contributes to improving mental and physical health. All adolescents and young people, including those that happen to have disabilities, experience benefits of leisure. As with any young person, young people with disabilities are a heterogeneous group with a diverse repertoire of needs depending on their characteristics, age, and stage of development.

The current concept of disability, and the degree to which people with a disability participate under equitable conditions, results from interaction between people and the environment (physical, social, and attitudinal). The extent to which people are impacted by their disability is determined by the capacity of each person to conduct tasks in different environments, along with the environmental elements that facilitate or limit their capacity (World Health Organization [WHO], 2001), and the needs and supports required by each person.

The study of the needs for leisure among young people with disabilities must be based on human rights, with the framework provided by: the International Convention on the Rights of Persons with Disabilities (United Nations [UN], 2006), the World Disability Report (World Health Organization [WHO] and Mundial Bank, 2011), the European Strategy on Disability 2010–2020 (European Union, 2010), the Spanish Integral Strategy for Culture for All (Government of Spain, 2011b), the Spanish Strategy on Disability 2012–2020 (Government of Spain, 2011a), and the General Law on the Rights of Persons with Disabilities and their Social Inclusion (Government of Spain, 2013). Accordingly, young people with disabilities demand access to, and active participation in, leisure based on equalitable opportunities. For this reason, it is necessary to promote positive and satisfactory experiences of inclusive leisure as a source of enjoyment and development in the community, for all citizens.

Research conducted in the last decade has studied the psychological, physical, and emotional benefits of leisure to different groups of people with intellectual or physical disability (Badia et al., 2013; King et al., 2013; Powrie et al., 2015). There has also been an increase in research supporting benefits of integration and inclusive in leisure, both for people with disabilities and those without disabilities (Lin-Ju Kang et al., 2010; Dattilo, 2018). Group leisure pursuits, in which people with and without disabilities participate, provide a context for all participants to increase self-esteem, improve acceptance of each other, and promote the creation and subsequent maintenance of friendship between children and young people with and without disabilities (Bowman et al., 2014; Bult et al., 2014).

More specifically, for people with disabilities, the benefits derived from leisure relate to more positive personal identity, greater personal adjustment, increased social skills, improved self-concept, more adaptive behavior, increased interactions and general improvement in skills (Kleiber et al., 2011; Kleiber and McGuire, 2016). Leisure provides an external stimulus that benefits the lives of people with disabilities in multiple areas (Sánchez and Rodríguez, 2008). It helps to develop physical, mental, emotional, and interpersonal capacities, such as generating greater self-confidence and improving socialization processes (Duquette et al., 2015). Larson et al. (2004) highlighted the positive consequences of leisure during adolescence: development of personal initiative, promotion of intrinsic motivation, acquisition of skills, respect for diversity and cultivation of responsibility. Furthermore, leisure provides social benefits such as harmony, cohesion, and positive social change (Ramos et al., 2012). It also provides personal benefits that enable fun, learning, mental development, psychological health and personal growth (self-identity and self-affirmation.

Finally, despite an array of studies (e.g., Jessup et al., 2013; Bowman et al., 2014; Law et al., 2015) highlighting the importance of leisure for young people with disabilities, these studies focus mainly on disability in general, or on a specific type of disability (hearing, physical, intellectual or visual). Few studies focus on participation in leisure, accounting for the effect of different types of disability.

The review of the literature shows that positive leisure is widely accepted as experience that contribute to development of autonomy, understood as the ability to self-organize, and to the well-being of young people in the transition to adulthood. However, leisure in the live of youth with disabilities is not well understood (Glendenning et al., 2003; Coleman, 2011).

Quantitative methods were used in this study to increase understanding of the leisure of young people with disabilities in the Basque region of northern Spain. In this text, we analyzed the relationship between self-managed leisure, understood the degree to which participants organized their leisure, with the satisfaction and perception of emotional, cognitive and behavioral benefits of young people with disabilities.

The aims of this work are threefold. First, to assess if gender and type of disability are associated with participation in self-managed leisure (organized by young people themselves). Second, to analyze if degree of participation in the organization of their own leisure influences satisfaction, and if there are variations according to gender and type of disability. Third, to assess if leisure independently organized by young people correlate with perceptions of psychological benefits (emotional, behavioral and cognitive).

## Methodology

### Population and Sample

For the current study, a representative sample was selected with the following parameters: sampling error of 4.8 sigmas, 95% confidence level, and the assumption that *p* = *q* = 0.5.

The sample consisted of 400 participants: 51.3% male and 48.8% females. We collected a stratified random sample of the population of young people with disabilities (15–29 years old) living in the Basque region of northern Spain. The sample was evenly distributed among the four types of disability (hearing, cognitive, physical and visual), 100 cases each.

### Instrument and Variables

For the purpose of this study, we created an *ad hoc* questionnaire, the aim of which was to assess the leisure of young people with disabilities and determinate how it relates relates to perceived benefits. The instrument was reviewed through the judgment of 20 experts in the field of leisure and disability, prestigious academics and researchers in leisure studies from various Spanish universities and several disciplines (social pedagogy, education, social psychology), as well as professionals with a long career in the third sector and the management of leisure services for people with disabilities were selected. The experts were informed of the group under study, the objectives and the hypotheses of the study, the questionnaire and the application instructions, accompanied by a script for its evaluation. The considerations made by the expert group were taken into account and the questionnaire was modified.

The questionnaire was validated through a pilot test with 150 young people with disabilities. During the pilot test, research team applied the questionnaire, taking into account the characteristics of the group under study, the objective was to test the functioning of the questionnaire in the field, that is, whether the wording of the questions was adequate for a good understanding of them, as well as to verify the time of application. A first descriptive and inferential analysis of the data was also carried out. The results of the pilot test suggested some modifications on the writing of some of the questions and the extension of the questionnaire, before carrying out the complete sampling.

This paper is based on six variables derived from the questionnaire:

1 – Individual responsibility for leisure. This variable assesses the participation of young people with disabilities in the organization of their own leisure (level 1 = self-managed) or whether other agents (peer group – level 2; family or association – level 3) organize them. A single response option was allowed for each of the eight recreation activities identified.
2 – Satisfaction with leisure. This variable assesses the degree of enjoyment with leisure. They identified their eight most important recreation activities and, then, they indicated the degree of satisfaction through a five-point Likert scale.
3 – Perception of the relationship between the enjoyment of an activity and individual responsibility for organizing their activities. This variable assesses the perception of the participants of whether greater level of participation in organizing each activity influences satisfaction with the activity. Responses were rated on a five-point Likert scale (1 = None, 2 = A little, 3 = More or less, 4 = A lot, and 5 = A lot).
4–6 – Positive impact of leisure. This comprised three dichotomous (yes, no) variables, which assess participants’ perception of emotional, cognitive, and behavioral benefits of their leisure.

### Field Work

The ethics committee of the University of Deusto evaluated the design of the research and provided necessary permits for its development. To recruit participants to this study, we identified all registered organizations for people with disabilities in the Basque region. We contacted the boards of directors for each entity, explained the purpose of the study, and guaranteed that data would be confidential and that all necessary permits were granted. Participation of the young people was voluntary, with legal guardian authorization required in the case of minors under the age of 18.

A member of the research team trained professionals from each disability organization to administer the questionnaire. The training lasted one day, during which the objective of the study, each question of the questionnaire and the instructions for its application (multiple answers, scales, support tables) were explained to the interviewers, followed by at least two application tests with each interviewer to check and correct the application mode. The professionals of the leisure services of the disability organization administered the questionnaire. The data collection process began in September 2016 and lasted until December 2016.

### Data Analysis

Several descriptive and inferential analyses were conducted to determine if stated objectives were achieved. For the first objective, frequencies of identified benefits were calculated, whether the activities were self-managed or not. Chi-square tests were calculated to test a possible relationship between the identified benefits and self-management.

With respect to the second objective, frequencies of sex at each level of self-management were calculated, as well as the frequencies of type of disability at each level of self-management. Chi-square tests were also calculated to test for a relationship between sex, on the one hand, and the type of disability, on the other, and self-management.

Finally, with regard to the third objective, the means and the standard deviations were calculated to determine mean levels of satisfaction with leisure at the different levels of self-management. To test for an effect of sex and type of disability, an analysis of variance was conducted, as well as testing the effect size for gender and Scheffé’s *post hoc* test for type of disability. Level of significance established for this study was *p* < 0.05.

## Results

The first hypothesis was that gender would determine their participation in leisure independently organized by young people (self-management). The second hypothesis stated that the type of disability of young people determine their participation in leisure independently organized by young people.

The data show that there was a relationship between gender and self-management of leisure (Table 1), with women reporting higher levels of self-management for leisure (self-managed-level 1) (84.1%) than men (72.3%). Similarly, there was a relationship between the type of disability and self-management of leisure (Table 1). Young people with physical disabilities reported higher levels of self-management (level 1) (88.1%).

**TABLE 1:** Participation in self-managed leisure activities depending gender and type of disability.

	Total	Self-managed activities		Activities organized by others		χ^2^	*p*
	
	*N*	*n*	%	*n*	%		
**Gender**							
Male	205	146	72.3	56	27.7	9.51	0.002
Female	195	164	84.1	31	15.9		
**Type of disability**							
Physical	101	89	88.1	12	11.9	8.85	0.031
Visual	100	73	73	27	27		
Hearing	100	75	75	25	25		
Intellectual	99	73	73.7	26	26.3		

*χ^2^ = Chi-square test; *p* = significance value.*

The third hypothesis stated that there would be a significant association between satisfaction with leisure and level of self-management, and that these results would vary according to gender and type of disability. Level of participation in organizing leisure was categorized into three levels. The first level corresponds to leisure independently organized by the young person (level 1 – self-management). The second level corresponds to leisure organized by peer group (level 2 – peer group). Finally, the third level corresponds to leisure in which the young person does not organize the activity, and family or disability association organizes the experiences.

In the analysis of the variation in satisfaction according to gender (Table 2), there were no significant differences between men and women, regardless of the degree to which the organized their leisure (level 1 – self-managed; peer group – level 2; family or association – level 3). High scores were observed at all levels, with the latter being quite similar, and indicating that gender does not differ in the levels of satisfaction, regardless of the degree to which they organized their leisure.

**TABLE 2:** Degree of satisfaction with leisure activities depending type of disability and degree of self-management.

	Total (*n* = 208)		Women (*n* = 107)		Men (*n* = 101)		*F*	g.l.	*p*	*d*
			
	M	DE	M	DE	M	DE				
Satisfaction (spanning 1–5) Level 1 – self-managed	3.98	1.10	3.97	1.15	3.98	1.05	0.01	1.206	0.962	0.01

	**Total (*n* = 164)**		**Women (*n* = 84)**		**Men (*n* = 80)**		***F***	**g.l.**	***p***	***d***
			
	**M**	**DE**	**M**	**DE**	**M**	**DE**				

Satisfaction (spanning 1–5) Level 2 – peer group	4.25	0.83	4.19	0.96	4.30	0.66	0.76	1.162	0.383	0.13

	**Total (*n* = 189)**		**Women (*n* = 99)**		**Men (*n* = 90)**		***F***	**g.l.**	***p***	***d***
			
	**M**	**DE**	**M**	**DE**	**M**	**DE**				

Satisfaction (spanning 1–5) Level 3 – family or disability association	4.19	0.89	4.11	0.93	4.28	0.85	0.01	1.187	0.206	0.19

*M = mean; DE = standard deviation; *F* = Snedecor’s *F*; g.l. = degrees of freedom; *p* = significance value; *d* = Cohen’s *d*.*

However, with regard to the association between satisfaction with leisure and type of disability, there were statistically significant differences depending on the degree to which they organized their leisure (Table 3). Therefore, the satisfaction of young people with disabilities with self-managed leisure differs according to the type of disability. Schefe’s *post hoc* test showed the visual disability group was significantly different from each of the other types of disabilities (*p* = ≤ 0.001 in all cases), with the effect size being very high in all cases (*d*_*visual–physical*_ = 1.18; *d*_*visual–hearing*_ = 1.19; *d*_*visual–intellectual*_ = 1.27).

**TABLE 3:** Degree of satisfaction with leisure activities depending type of disability and degree of self-management.

	Total (*n* = 208)		Physical disability (*n* = 54)		Visual disability (*n* = 46)		Hearing disability (*n* = 64)		Intellectual disability (*n* = 44)		*F*	g.l.	*p*
					
	M	DE	M	DE	M	DE	M	DE	M	DE			
Satisfaction (spanning 1–5) Level 1 – self-managed	3.98	1.10	4.28	0.90	2.96	1.35	4.17	0.72	4.38	0.83	22.35	3.204	≤0.001

	**Total (*n* = 164)**		**Physical disability (*n* = 38)**		**Visual disability (*n* = 21)**		**Hearing disability (*n* = 72)**		**Intellectual disability (*n* = 33)**		***F***	**g.l.**	***p***
					
	**M**	**DE**	**M**	**DE**	**M**	**DE**	**M**	**DE**	**M**	**DE**			

Satisfaction (spanning 1–5) Level 2 – peer group	4.25	0.83	4.41	0.80	4.13	0.94	4.22	0.66	4.22	1.08	0.66	3.160	0.574

	**Total (*n* = 167)**		**Physical disability (*n* = 50)**		**Visual disability (*n* = 23)**		**Hearing disability (*n* = 66)**		**Intellectual disability (*n* = 50)**		***F***	**g.l.**	***p***
					
	**M**	**DE**	**M**	**DE**	**M**	**DE**	**M**	**DE**	**M**	**DE**			

Satisfaction (spanning 1–5) Level 3 – family or disability association	4.19	0.89	4.17	1.01	4.18	0.86	4.13	0.78	4.32	0.93	0.44	3.185	0.720

*M = mean; DE = standard deviation; *F* = Snedecor’s *F*; g.l. = degrees of freedom; *p* = significance value; *d* = Cohen’s *d*.*

Regarding leisure organized by the peer group (level 2) and by external agents (level 3), there were significantly different scores in the satisfaction among the different types of disability (Table 3). For example, in the activities managed by the peer group, the average level of satisfaction among young people with physical disabilities was considerably higher than the average for the total sample (*m*_*physical*_ = 4.41/*m*_*total*_ = 4.25).

There were no statistically significant differences between different types of disability. Therefore, if the peer group and/or external agents manage the leisure the levels of satisfaction did not differ according to type of disability. Therefore, regarding the third hypothesis, there was a significant association between degree of satisfaction with leisure and the level of self-management of these experiences, and that these results varied by type of disability but not gender.

The final hypotheses stated that the level of self-management of leisure would determine participants’ perception of psychological benefits. Three types of benefits were assessed: emotional, behavioral, and cognitive.

Results showed (Table 4) significant relationships between perceived emotional and cognitive benefits and level of self-management of leisure. Perceived cognitive benefits showed the strongest relationship with self-management of leisure (χ^2^ = 40.88; *p* = ≤ 0.001). In the case of perceived behavioral benefits, no significant relationship with self-management was found (χ^2^ = 2.81; *p* = 0.60).

**TABLE 4:** Perception of psychological benefits depending the degree of self-management of leisure activities.

	Total	Self-managed activities		Activities organized by others		χ^2^	*p*
	
	*N*	*n*	%	*n*	%		
**Psychological benefits**							
**Emotional**							
Yes	330	272	87.7	58	64.4	26.22	≤0.001
No	70	38	12.3	32	35.6		
**Behavioral**							
Yes	78	66	21.3	12	13.3	2.81	0.060
No	322	244	78.7	78	86.7		
**Cognitive**							
Yes	185	170	54.8	15	16.7	40.88	≤0.001
No	215	140	45.2	75	83.3		

*χ^2^ = Chi-square test; *p* = significance value.*

Based on the results, it is possible to accept the hypothesis that the degree to which young people organized their leisure influences their perception of psychological benefits, although only in the case of emotional and cognitive benefits, not so in the case of behavioral benefits.

## Discussion

This study demonstrates that both gender and type of disability correlate with self-management of leisure. Young women with disabilities reported a higher level of self-management than young men with disabilities did. In addition, people with physical disabilities reported greater self-management of their leisure compared to people with visual, hearing, or intellectual disabilities. For leisure with greatest degree of self-management, gender was not correlated with level of satisfaction. On the other hand, the type of disability was related to levels of satisfaction.

In the same way that, for several decades, leisure services for other young people have incorporated processes in which young people decide and organize their leisure, entities of people with disabilities should include these same participatory processes since they generate emotional benefits, in the satisfaction with their leisure, and provide them with capacities for decision making. In the study, some results vary according to gender and type of disability, so these variables must be considered in these processes.

To solve this situation Arellano and Peralta (2016), propose the application of Person Centered Planning (PCP) in the field of education and leisure, as a paradigm of inclusion improving the self-determination of people with disabilities (Brown et al., 2007). Arellano and Peralta (2016) apply this method to people with intellectual disabilities, since they consider that it is in the groups with greater difficulties of autonomy where the greatest benefits can be extracted, as well as in other groups with specific needs.

Results of this study reveal that, among young people with disabilities in the Basque region of Spain, the level of satisfaction with their leisure is high. However, level of satisfaction differs depending on the level of self-management of leisure, when young people organize their leisure, they show higher levels of satisfaction. These results are consistent with previous findings that the social and emotional development of young people is linked to the capacity for autonomous action (Ryan, 1993; Deci, 1995; Brandtstadter, 1998) and that leisure offers opportunities to acquire greater autonomy and self-management (Ortega et al., 2015).

Therefore, it would make sense to strengthen the provision of inclusive and person-centered public and private leisure as a substantial element in the development of the autonomy of people with disabilities, considering that it is not a sole group, but instead each person has different needs that must be taken into account in the design of leisure offerings. Furthermore, it is necessary to include information and data collection elements to improve inclusion in educational and leisure projects, as proposed by Booth and Ainscow (2011).

Finally, we can confirm the importance of including gender and type of disability in studies on the benefits of leisure people with disabilities. Both gender and type of disability were associated with satisfaction with leisure, and higher satisfaction was associated with greater perceived benefits of leisure. These findings are consistent with numerous studies indicating that leisure have a positive effect on emotional development and self-determination (Osgood et al., 1996; Larson, 2000; Leversen et al., 2012; McDonough et al., 2013; King and Church, 2015; Anderson, 2017).

Many studies have analyzed leisure and their benefits among people with disabilities (Lin-Ju Kang et al., 2010; Bowman et al., 2014; Bult et al., 2014; Kleiber and McGuire, 2016; Dattilo, 2018). A contribution of this study is the finding that, in the case of young people with visual disabilities, their degree of satisfaction was significantly lower than young people with physical, hearing or intellectual disabilities, after taking into account the leisure with a greater degree of self-management. Furthermore, this leisure is associated with the perception of emotional and cognitive benefits. It is necessary to delve into the origin of this difference and, consequently, incorporate new methods such as Person Centered Planning in the education of the persons with visual disabilities and assess whether it improves their satisfaction with self-managed activities.

Finally, on the one hand, it can therefore be stated that leisure, both organized by young people and by other agents, generates benefits, mainly emotional and cognitive, thanks to the interaction with other people with and without disabilities. On the other hand, it is important to design and develop leisure offerings that improve and advance achieving behavioral benefits among the group of young people with disabilities, as a step forward in the provision of services.

The present study has several limitations that should be addressed. First, the results do not allow us to make causal conclusions about the relationships between the variables (degree of self-management, benefits and satisfaction), although our findings support the possibility of such a relationship. Future studies could be conducted to further investigate the causality of the relationships identified in the present work paper. Second, the sample size does not allow to generalize the results by type of disability. Thus, in future studies, larger samples could be used in each type of disability.

## Data Availability Statement

The datasets generated for this study are available on request to the corresponding author.

## Ethics Statement

The studies involving human participants were reviewed and approved by the ethics committee of the University of Deusto. Written informed consent to participate in this study was provided by the participants’ legal guardian/next of kin.

## Author Contributions

IL and AM conceived the objective of the work and the methodological design. JD, IL, and AM designed the tool and participated in the validation. JD contributed to the data collection and analysis of the results. JD, IL, and AM were responsible for writing the manuscript and critically reviewing it for important intellectual content, all authors discussed the results and contributed to the final manuscript.

## Conflict of Interest

The authors declare that the research was conducted in the absence of any commercial or financial relationships that could be construed as a potential conflict of interest.
